# Cuproptosis patterns and tumor immune infiltration characterization in colorectal cancer

**DOI:** 10.3389/fgene.2022.976007

**Published:** 2022-09-13

**Authors:** Yan Du, Yilin Lin, Bo Wang, Yang Li, Duo Xu, Lin Gan, Xiaoyu Xiong, Sen Hou, Shuang Chen, Zhanlong Shen, Yingjiang Ye

**Affiliations:** ^1^ Department of Gastroenterological Surgery, Peking University People’s Hospital, Beijing, China; ^2^ Laboratory of Surgical Oncology, Peking University People’s Hospital, Beijing, China; ^3^ Key Laboratory of Colorectal Cancer Diagnosis and Treatment Research, Beijing, China

**Keywords:** CRC, cuproptosis, immune status, overall survival, gene signature

## Abstract

Faced with the high heterogeneity and poor prognosis of colorectal cancer (CRC), this study sought to find new predictive prognostic strategies to improve the situation. Cuproptosis is a novel cell death mechanism that relies on copper regulation. However, the role of cuproptosis-related gene (CRG) in CRC remains to be elucidated. In this study, we comprehensively assessed the CRG landscape in CRC based on The Cancer Genome Atlas (TCGA). We identified differential expression and genetic alterations of CRG in CRC. CRG is highly correlated with initiation, progression, prognosis, and immune infiltration of CRC. We construct a risk score signature containing 3 CRGs based on LASSO. We explored the correlation of CRG-Score with clinicopathological features of CRC. Age, stage, and CRG-Score were integrated to construct a nomogram. The nomogram has robust predictive performance. We also understand the correlation of CRG-Score with CRC immune landscape. CRG-Score can effectively predict the immune landscape of CRC patients. Low-risk CRC patients have greater immunogenicity and higher immune checkpoint expression. Low-risk CRC patients may be better candidates for immunotherapy. At the same time, we also predicted more sensitive drugs in the high-risk CRC patients. In conclusion, the CRG risk score signature is a strong prognostic marker and may help provide new insights into the treatment of individuals with CRC.

## Introduction

Colorectal cancer (CRC) shows a steady upward trend worldwide, and its morbidity and mortality ranks third among all malignant tumors (Siegel et al., 2022). Despite advances in treatment and diagnosis in recent years, a mass of patients still die from cancer recurrence and metastasis. The 5-years survival rate is only 14.0% (Olenius et al., 2022). This is often attributed to the high degree of tumor heterogeneity and complex dynamic evolution (Hanahan and Weinberg, 2011). Therefore, more prognostic-related factors are needed for precise risk stratification of patients. To guide a more effective and individualized treatment plan.

Copper is an indispensable nutrient in the human body as a cofactor for essential enzymes. However, dysregulation of copper homeostasis may also lead to many diseases (Oliveri, 2022). Extensive research shows that dysregulation of copper homeostasis plays a key role in cancer initiation and progression (Shanbhag et al., 2021). Currently, significant abnormalities in copper content have been found in serum and tumor tissues of different cancers (gallbladder, breast, thyroid, colorectal, lung, and oral) (Basu et al., 2013; Ding et al., 2015; Baltaci et al., 2017; Stepien et al., 2017; Zhang and Yang, 2018; Chen et al., 2019; Aubert et al., 2020). At the same time, high levels of copper are associated with higher stages of colorectal and breast cancer (Gupta et al., 1993; Denoyer et al., 2015). Copper can promote tumor progression and metastasis by activating fibroblast growth factor 1, angiopoietin, interleukin 1 and vascular endothelial growth factor (Lelièvre et al., 2020; Li, 2020). Based on the above mechanisms, copper chelators (elesclomol, disulfiram, and dithiocarbamates) and copper ion carriers (trientine, tetrathiomolybdate) have been used in carcinoma treatment and have been shown to be effective against cancer stem cells (Brady et al., 2017; Davis et al., 2020; Chen et al., 2006; O'Day et al., 2013). Recently, researchers discovered a new copper-dependent and copper-regulated cell death mechanism called Cupproptosis. Copper binds to proteins containing fatty acylated structures in the tricarboxylic acid (TCA) cycle, resulting in abnormal aggregation of the latter and loss of iron-sulfur cluster proteins, triggering proteotoxic stress and eventual cell death (Tsvetkov et al., 2022). However, cancer metastasis is highly dependent on TCA cycle reprogramming. Downregulation of the TCA cycle releases CO_2_, lactate, and other organic acids to benefit tumor invasion (Faubert et al., 2020). At the same time, the altered microenvironment suppresses the activation of immune cells and promotes immune escape (Cerezo and Rocchi, 2020). At present, some genes that can regulate cuproptosis have been identified. However, the clinical impact of cuproptosis-related gene (CRG) on CRC still needs to be further elucidated. This may help to accurately predict the prognosis of CRC patients.

Transcriptome data of 612 CRC samples from TCGA database were collected in this study. We collected 10 CRGs from previous studies (Tsvetkov et al., 2022). We identified differential expression and genetic alterations of CRG in CRC. CRG is highly correlated with initiation, progression, prognosis, and immune infiltration of CRC.

We successfully constructed a CRG risk score signature to quantify cuproptosis levels in individual tumors. The nomogram integrating the CRG-Score has robust predictive performance. It can help patients accurately determine survival outcomes. We found that the CRG-Score could effectively predict the immune landscape of CRC patients. And predict the sensitivity of different CRG-Score patients to immunological drugs and chemical drugs. In conclusion, the CRG risk score signature is a strong prognostic marker and may help provide new insights into the treatment of individuals with CRC.

## Materials and methods

### Data collection

TCGA database (https://portal.gdc.cancer.gov/repository) accessed: 27 May 2022. GEO database (https://www.ncbi.nlm.nih.gov/geo/) Accessed: 9 August 2022. The study consisted of 1412 CRC data from 5 cohorts (TCGA-COAD, TCGA-READ, GSE17538, GSE29623, GSE39582). The genes transcriptome expression profile of CRC patients was obtained from the above 5 cohorts. TCGA expression data were converted to fragment per kilobase million (FPKM) values prior to use. The “affy” and “simpleaffy” packages were used to normalize GEO data. The dataset was batch corrected using combat in the “sva” package. DNA mutation data of CRC patients were obtained from TCGA-COAD cohort and TCGA-READ cohort. Clinical information was obtained from the respective matched cohorts. To reduce statistical errors in the analysis, we excluded CRC patients with short overall survival (OS) values (<30 days) and missing information. According to the ratio of 1:1, TCGA CRC patients were randomly divided into train and test groups.

### Construction of a cuproptosis-related gene signature

10 CRGs retrieved from previous reports (Tsvetkov et al., 2022). A detailed list of CRGs is shown in the attached file: Supplementary Table S1. We screen the CRG using iterative LASSO (Least Absolute Shrinkage and Selection Operator) with 1,000 iterations. To prevent overfitting, for each iteration, 1,000 random stimuli were set. The area under the curve (AUC) was calculated from the receiver operating characteristic curve (ROC). The inclusion was stopped when the AUC reached its peak, and the obtained CRG was used to establish the CRG risk score signature. CRG-Score=(mRNA1 expression × coefficient mRNA1) + (mRNA2 expression × coefficient mRNA2) + (mRNA3 expression × coefficient mRNA3). CRC patients were divided into low/high risk groups according to the median value of CRG-Score.

### Functional enrichment and immune correlation analysis

The Kyoto Encyclopedia of Genes and Genomes (KEGG) analysis on Gene Set Enrichment Analysis (GSEA) software (version 4.2.3) was used to assess pathway activity. CIBERSORT-ABS, CIBERSORT, EPIC, MCPcounter, QUANTISEQ, TIMER, and XCELL on TIMER2.0 were used to assess immune infiltration status. Immune-related pathway activity was assessed using the Single-sample gene set enrichment analysis (ssGSEA) algorithm. Tumor mutational burden (TMB) score analysis was performed between CRG-Score risk groups. Expression levels of 47 immune checkpoint-related genes (He et al., 2022) were analyzed between CRG-Score risk groups.

### Tumor Immune Dysfunction Exclusion

Data on clinical response to immune checkpoint inhibitors in CRC are lacking. We used the Tumor Immune Dysfunction Exclusion (TIDE) website to predict efficacy between CRG-Score risk groups. The resulting data can be obtained after uploading the expression profile data to the TIDE website (https://tide.dfci.harvard.edu) (Lu et al., 2019).

### Drug susceptibility prediction

To help clinical patients achieve better drug outcomes. Referring to Genomics of Drug Sensitivity in Cancer (GDSC, https://www.cancerrxgene.org), sensitivity to common chemotherapeutics and targeted drugs was predicted between CRG-Score risk groups. The evaluation index is the median inhibitory concentration (IC50).

### Statistical analysis

R software (version 4.1.2) was used for analysis and plotting of all data in this study. The “caret” package is used to randomize groupings. The “limma” package is used to extract the expression levels of CRGs in downloaded mRNA expression profiles. The “ggpubr” and “reshape2”packages are used to draw boxplots. The “maftools” package is used to draw waterfall charts. The “RCircos” package is used to draw circle diagrams. The “forestplot” package is used to draw forest plots. The “timeROC” package is used for ROC. The “scatterplot3d” package is used for principal components analysis (PCA). The “survival” and “survminer” packages are used to draw survival curves. The “pheatmap” package is used to plot survival status, risk heatmaps and risk curves. The “regplot” and “rms” packages are used to draw nomograms and calibration curves. The “clusterProfiler” package is used for gene ontology (GO) analysis. The “pRRophetic” package was used for drug susceptibility comparisons.

Strawberry Perl software was used for data processing. The illustrations by Figdraw. Statistical differences between the two groups were compared using the Kruskal-Wilcoxon test. The Kruskal–Wallis test was used to compare statistical differences among three or more groups. All statistical *p*-values are two-way outcomes. Only *p* < 0.05 was considered statistically significant.

## Results

### Landscape of Cuproptosis-related Gene in colorectal cancer

**FIGURE 1:**
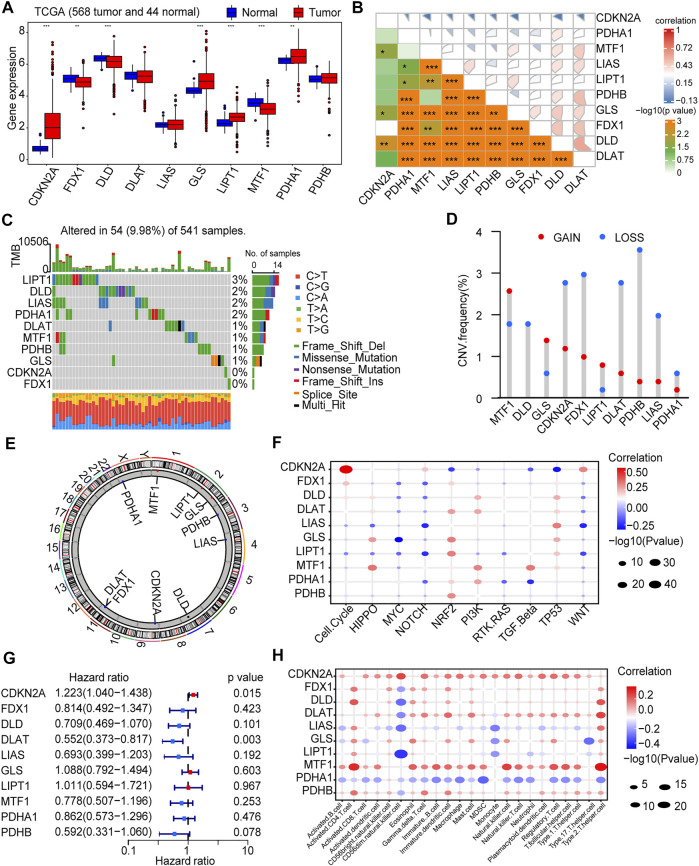
Landscape of Cuproptosis-related Genes (CRGs) in CRC. **(A)** Expression of CRGs in colorectal tumor tissues and adjacent non-tumor tissues from TCGA-COAD and TCGA-READ (612 patients: 568 tumor and 44 normal). **(B)** Correlation between CRGs expression. **(C)** Gene mutation of CRGs. **(D)** Copy number variation (CNV) frequency of CRGs. **(E)** The location on the chromosome where CRGs CNV changes. **(F)** Correlation between CRGs and CRC important initiation and progression mechanisms. **(G)** Univariate COX regression analysis of the hazard ratio between CRGs and CRC overall survival. **(H)** Correlation of CRGs and immune cell infiltration. *p < 0.05, **p < 0.01, ***p < 0.001.

Based on previous literature reports, we included a total of 10 CRGs (CDKN2A, DLAT, DLD, FDX1, GLS, LIAS, LIPT1, MTF1, PDHA1, and PDHB) (Tsvetkov et al., 2022) for study. We first explored CRG expression changes in mRNA expression profiles. The results showed that most of the CRGs (7/10, 70%) were differentially expressed between tumor tissues and adjacent non-tumor tissues (*p* < 0.05). CDKN2A, GLS, LIPT1, and PDHA1 were up-regulated in tumor samples, and FDX1, DLD, MTF1 were down-regulated in tumor samples (Figure 1A). Furthermore, there was a strong association between these genes (Figure 1B). Subsequently. We performed SNV and CNV analyses based on data from the TCGA-COAD cohort and the TCGA-READ cohort. The results showed that only 54 (9.98%) of 541 CRC samples had CRGs mutations (Figure 1C), and the mutation frequency was very low. CNV alterations are not prevalent in these genes. MTF1 had the most significant copy number gain, while PHDB exhibited the most significant copy number loss (Figure 1D). The chromosomal location changes of CRGs CNVs are shown in the figure (Figure 1E).

The role of CRGs in CRC is currently unclear. We analyzed the correlation between CRGs and important initiation and progression mechanisms of CRC. CRG was strongly associated with important initiation and progression mechanisms of CRC (Figure 1F). The relationship between CRGs and prognosis of CRC patients was further explored. Univariate COX regression analysis showed that CDKN2A was a risk factor for OS (*p* < 0.05) and DLAT was a protective factor for OS ((*p* < 0.05, Figure 1G; Supplementary Table S2). Finally, there is growing evidence that the tumor immune landscape is closely related to tumor prognosis and treatment outcomes (Chen and Mellman, 2017). Therefore, we further explored the relationship between CRGs and cellular infiltration in CRC. The expression level of most CRGs strongly correlated with the level of immune cell infiltration (*p* < 0.05, Figure 1H).

### Construction and evaluation of the Cuproptosis-related Gene risk score signature

**FIGURE 2:**
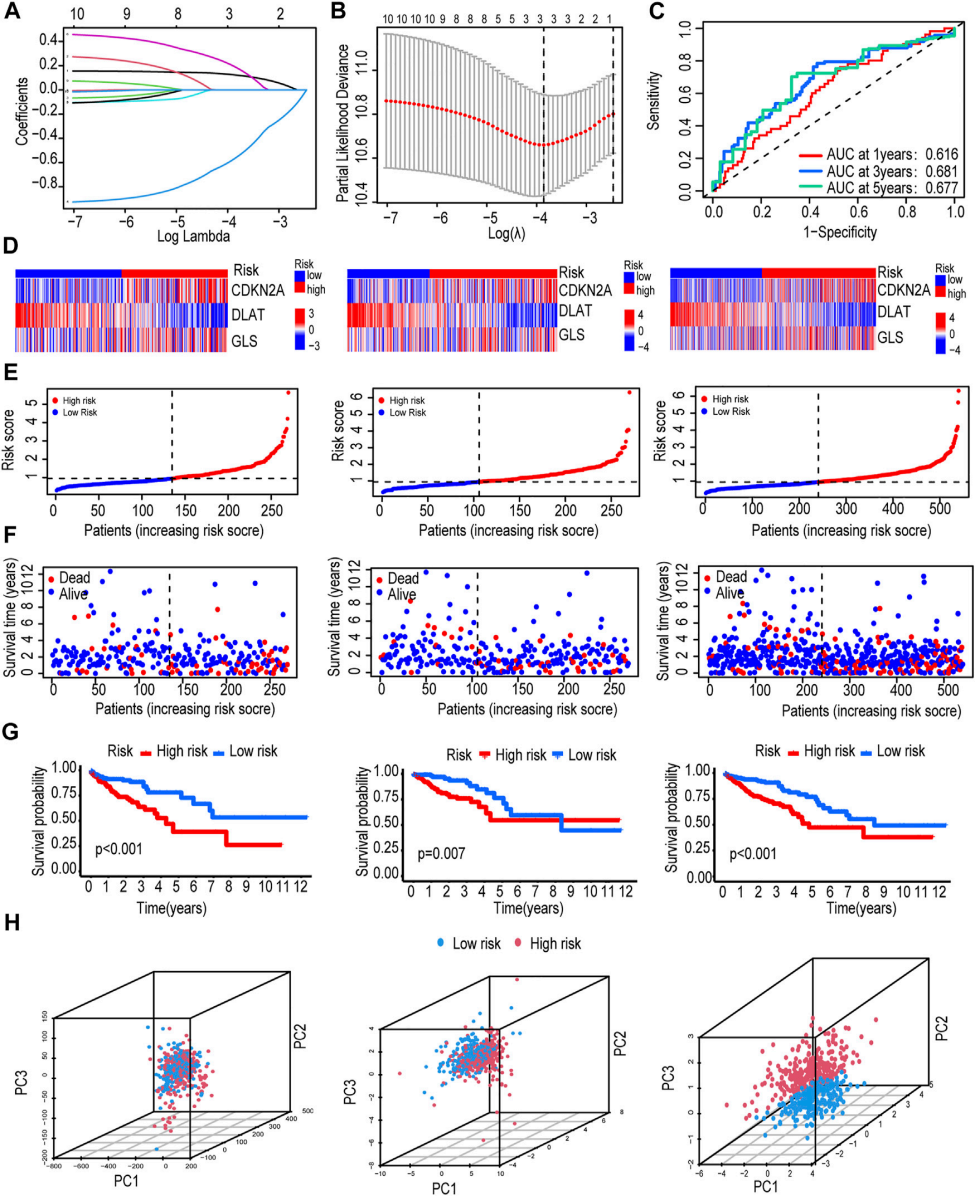
Construction and evaluation of the CRG risk score signature. **(A,B)** Use iterative LASSO to construct a CRG risk score signature. **(C)** Time-dependent receiver operating characteristic (ROC) curve validated the prognostic performance of CRG-Score. **(D)** Heatmap of the expression of 3 CRGs in train group, test group and total group. **(E)** CRG-Score distribution in train group, test group and total group. **(F)** CRG-Score survival status in train group, test group and total group. **(G)** Survival time between CRG-Score groups in train group, test group and total group. **(H)** Principal component analysis (PCA).

According to the ratio of 1:1, 540 CRC patients were randomly divided into train and test groups. The detailed clinical information of the test group, train group and total group is shown in Table 1. Based on 10 CRGs described above, we used iterative LASSO to construct a CRG risk score signature for predicting CRC survival. And 3 genes were extracted when the first-order value of Log(λ) was the minimum likelihood of bias (Figures 2A,B).

**TABLE 1:** Clinical information of train, test, total groups.

Covariates	Total	Test	Train	*p* value
Age				0.3854
age≤65	235 (43.52%)	123 (45.56%)	112 (41.48%)	
age>65	305 (56.48%)	147 (54.44%)	158 (58.52%)	
Gender				0.7301
FEMALE	253 (46.85%)	129 (47.78%)	124 (45.93%)	
MALE	287 (53.15%)	141 (52.22%)	146 (54.07%)	
Stage				0.232
I	93 (17.22%)	49 (18.15%)	44 (16.3%)	
II	207 (38.33%)	111 (41.11%)	96 (35.56%)	
III	148 (27.41%)	73 (27.04%)	75 (27.78%)	
IV	77 (14.26%)	31 (11.48%)	46 (17.04%)	
unknown	15 (2.78%)	6 (2.22%)	9 (3.33%)	
T stage				0.8603
T1	15 (2.78%)	8 (2.96%)	7 (2.59%)	
T2	93 (17.22%)	48 (17.78%)	45 (16.67%)	
T3	368 (68.15%)	184 (68.15%)	184 (68.15%)	
T4	63 (11.67%)	30 (11.11%)	33 (12.22%)	
Tis	1 (0.19%)	0 (0%)	1 (0.37%)	
N stage				0.4122
N0	317 (58.7%)	166 (61.48%)	151 (55.93%)	
N1	129 (23.89%)	62 (22.96%)	67 (24.81%)	
N2	93 (17.22%)	42 (15.56%)	51 (18.89%)	
unknown	1 (0.19%)	0 (0%)	1 (0.37%)	
M stage				0.0896
M0	401 (74.26%)	204 (75.56%)	197 (72.96%)	
M1	76 (14.07%)	30 (11.11%)	46 (17.04%)	
unknown	63 (11.67%)	36 (13.33%)	27 (10%)	

**TABLE 2:** Clinical information of the high CRG-Score and low CRG-Score groups.

Covariates	High CRG-Score	Low CRG-Score	*p* value
Age			0.2477
age≤65	123 (41.14%)	112 (46.47%)	
age>65	176 (58.86%)	129 (53.53%)	
Gender			0.5338
Female	136 (45.48%)	117 (48.55%)	
Male	163 (54.52%)	124 (51.45%)	
Stage			0.0389
I	45 (15.05%)	48 (19.92%)	
II	106 (35.45%)	101 (41.91%)	
III	91 (30.43%)	57 (23.65%)	
IV	50 (16.72%)	27 (11.2%)	
unknown	7 (2.34%)	8 (3.32%)	
T stage			0.0318
T1	4 (1.34%)	11 (4.56%)	
T2	48 (16.05%)	45 (18.67%)	
T3	203 (67.89%)	165 (68.46%)	
T4	43 (14.38%)	20 (8.3%)	
Tis	1 (0.33%)	0 (0%)	
N stage			0.0019
N0	157 (52.51%)	160 (66.39%)	
N1	78 (26.09%)	51 (21.16%)	
N2	64 (21.4%)	29 (12.03%)	
unknown	0 (0%)	1 (0.41%)	
M stage			0.114
M0	216 (72.24%)	185 (76.76%)	
M1	49 (16.39%)	27 (11.2%)	
unknown	34 (11.37%)	29 (12.03%)	

The CRG risk score signature formula:CRG Score= (CDKN2A ×0.1649) - (DLAT ×08,399) + (GLS ×0.4064). CRC patients were divided into low/high risk groups according to the median value of CRG-Score (Table 2). AUC values were evaluated by ROC curve. The AUC values of the CRG risk score signature reached 0.616, 0.681, and 0.677 in the 1st, 3rd, and 5th years, respectively (Figure 2C). The expression of risk model genes for high-risk and low-risk patients in the train, test, and total groups is shown in a heatmap (Figure 2D). Comparison of risk score distribution, survival time and survival status among risk groups in the train, test, and total groups confirmed that high CRG-Score CRC patients had a worse prognosis (Figures 2E–G).

Among the 3 expression profiles (total gene expression profile, CRG expression profile, expression profile of 3 risk model genes), we used PCA to verify differences between CRG-Score risk groups. The 3 risk model genes had the best discriminative power, which could well distinguish high/low risk groups (Figure 2H).

In order to avoid analysis bias caused by a single database. We revalidated the CRG score signature by integrating 3 sets of CRC data from the GEO database (GSE17538, GSE29623, GSE39582). The expression of risk model genes for patients in the high-risk and low-risk groups is shown in a heat map (Supplementary Figure S1A). Risk score distribution, survival status, and survival time (Supplementary Figures S1B–D) reconfirmed that high CRG-Score CRC patients had a worse prognosis. The combination of the 3 risk model genes had the highest prediction accuracy with an AUC value of 0.633 (Supplementary Figure S1E).

### Clinicopathological features and biological functions between Cuproptosis-related Gene-Score groups

**FIGURE 3:**
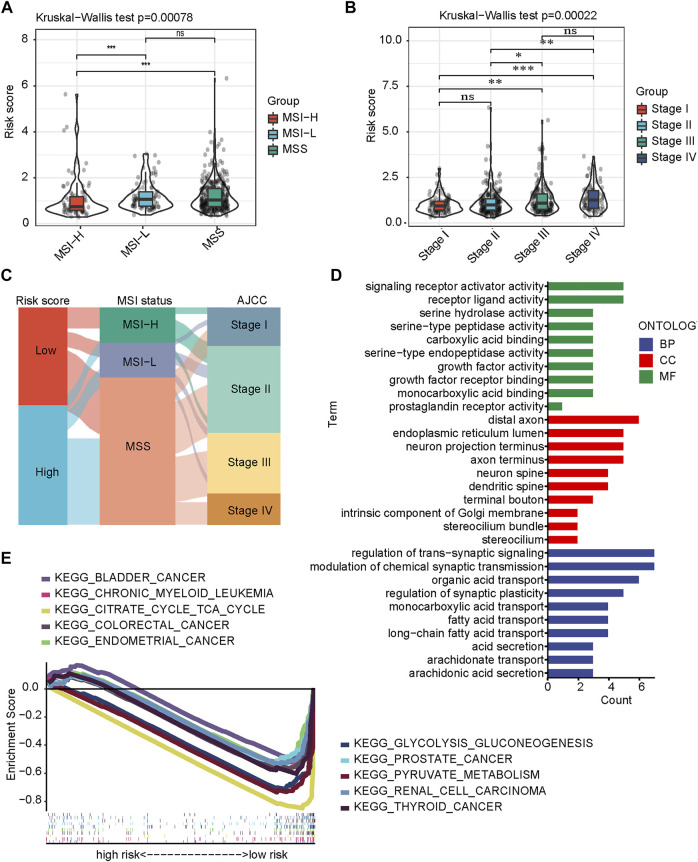
Clinicopathological features and biological functions between CRG-Score groups. **(A)** Differences in CRG-Score among CRC molecular subtypes (Kruskal–Wallis test). **(B)** Differences in CRG-Score in clinical staging of CRC (Kruskal–Wallis test). **(C)** Association of CRG-Score, molecular subtypes and clinical stage in CRC. **(D)** GO analysis. **(E)** KEGG analysis on GSEA.

To further validate the importance of CRG-Score in clinical practice, we examined its correlation with clinicopathological features. We first classified CRC into 3 subtypes: microsatellite stable (MSS), microsatellite low instability (MSI-L), and microsatellite high instability (MSI-H). The CRG-Score was significantly lower in the MSI-H subtype than in the MSS subtype (*p* = 0.00035) and MSI-L subtype (*p* = 0.0009, Figure 3A). This is consistent with current literature reports: MSI-H subtype has the best prognosis (Popat et al., 2005). In addition, the Wilcoxon test was used to compare different stages and high CRC scores were associated with high stages (Figure 3B). Interestingly, there was a stepwise increase in CRC score between clinical stages I and II, and between clinical stages III and IV, but lack of statistical significance (Figure 3B). The relationship between CRG-Score and CRC subtype and stage was visualized using a Sankey diagram (Figure 3C). These results suggest that the CRG-Score is able to characterize some clinical features and molecular subtypes of CRC patients.

We further explored differences in biological function between risk groups. GO analysis showed that signaling receptor activity, growth factor activity, and serine proteases activity were significantly enriched (Figure 3D). Growth and metabolic regulation that predict differences between risk groups. KEGG analysis showed that glycolysis-related pathways (pyruvate metabolism, glycolysis/gluconeogenesis, citric acid cycle) and some tumor-related pathways were significantly enriched (Figure 3E).

### Development and evaluation of nomograms

**FIGURE 4:**
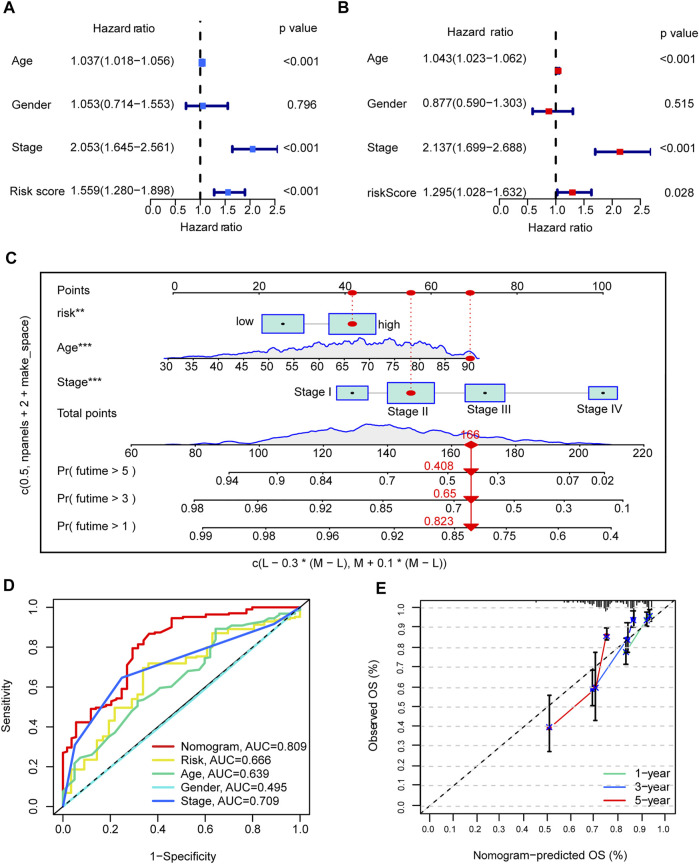
Development and evaluation of nomograms. **(A,B)** univariate and multivariate Cox analyses of CRG risk score and clinical information with overall survival. **(C)** nomogram. **(D)** The AUC value of Nomogram in the ROC curve is 0.809. **(E)** Calibration plots illustrate nomogram with excellent predictive power at 1st, 3rd and 5th years.

To build a CRC patient survival prediction model for clinical use, we first performed univariate and multivariate Cox regression. CRG-Score is an independent prognostic factor for OS. In univariate Cox, hazard ratio (HR) of the CRG-Score was 1.558 and 95% confidence interval (CI) of the CRG-Score was 1.280–1.898 (*p* < 0.001, Figure 4A; Supplementary Table S3). In multivariate COX, HR of the CRG-Score was 1.295 and 95% CI was 1.028–1.632 (*p* = 0.028, Figure 4B; Supplementary Table S3).

In addition, clinical tumor stage and age were also independent prognostic factors. We combined age, tumor stage and CRG-Score to graphically construct the final nomogram (Figure 4C). By calculating the score for each variable, a vertical line can be drawn to easily estimate the 1-, 3-, and 5-years survival of individual CRC patients. The ROC showed that the nomogram had excellent accuracy in terms of OS, AUC = 0.809 (Figure 4D). Meanwhile, the calibration plots illustrate that the nomogram achieves good agreement between the observed and predicted OS outcomes at 1st, 3rd and 5th years (Figure 4E).

### Correlation between Cuproptosis-related Gene-Score groups and immunity

**FIGURE 5:**
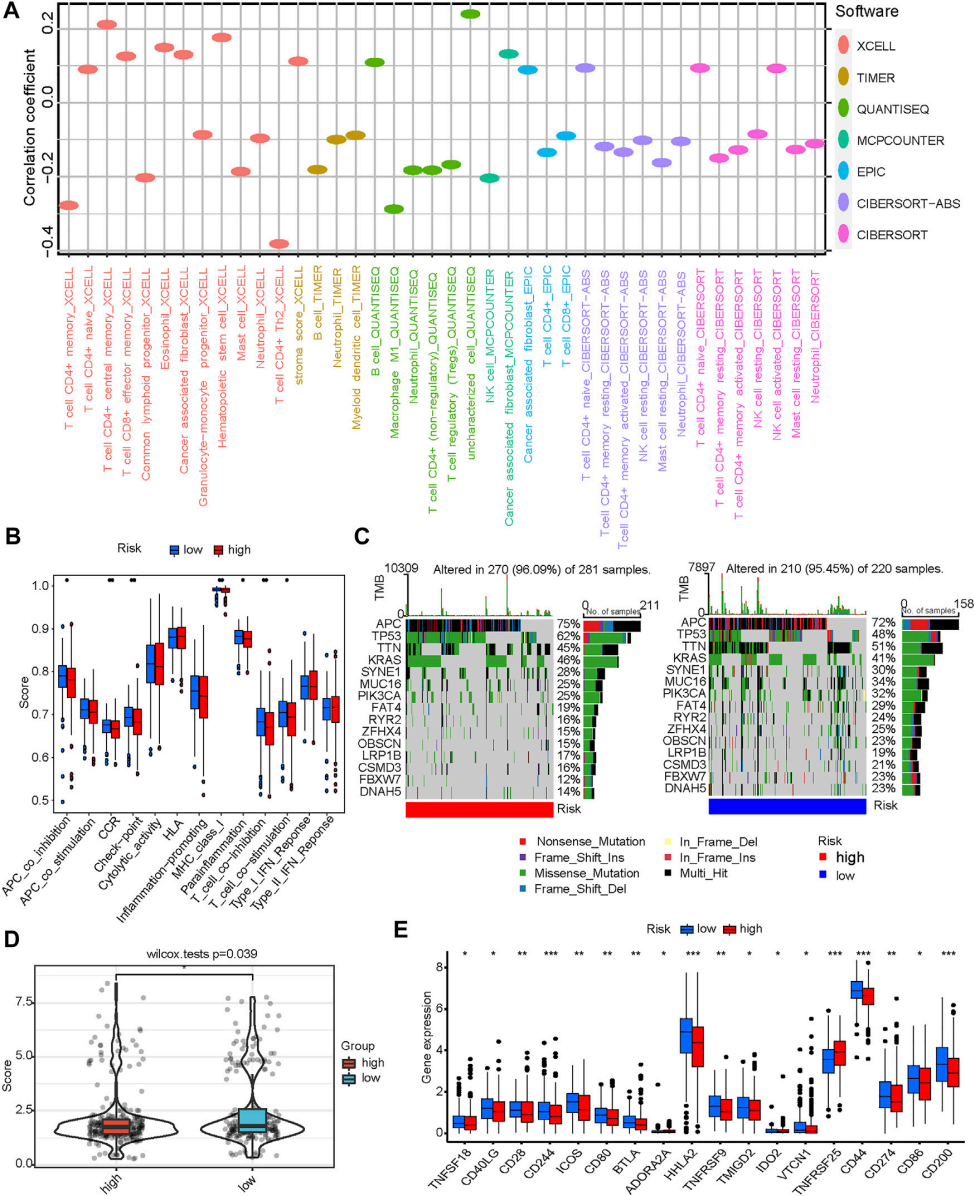
Correlation between CRG-Score groups and immunity. **(A)** Correlation between CRG-Score groups and immune infiltration status. **(B)** Correlation between CRG-Score groups and immune-related pathway activity. **(C)** Oncoplot represents the top 15 mutated genes between CRG-Score groups. **(D)** Tumor mutational burden (TMB) between CRG-Score groups. **(E)** Correlation between CRG-Score groups and expression levels of immune checkpoint-related genes. *p < 0.05, **p < 0.01, ***p < 0.001.

CRG-Score plays an excellent role in predicting prognosis, and we next explored differences in immune signatures between CRG-Score risk groups in CRC and their potential value in guiding individualized treatment. We first assessed immune infiltration status using several different platforms (CIBERSORT-ABS, CIBERSORT, EPIC, MCPcounter, QUANTISEQ, TIMER and XCELL) with a filter criterion of *p* < 0.05. Immune cell bubble plot showed: T-cells CD4^+^, NK cells, macrophage M1, myeloid dendritic cells were associated with the CRG-Score low risk group (Figure 5A). Hematopoietic stem cells and cancer-associated fibroblasts were associated with the CRG-Score high risk group (Figure 5A). The CRG-Score low risk group has a higher immune infiltration status and the CRG-Score high risk group has more stromal cells.

Next, immune-related pathway activity was assessed using ssGSEA with a filter criterion of *p* < 0.05. The results showed that the MHC class I, CCR, Checkpoint, Parainflammation and T cell co-stimulation scores were significantly lower in CRG-Score high risk group than in CRG-Score low risk group (Figure 5B). This is consistent with the immune infiltration results described above, suggesting a higher immunogenicity in CRG-Score low risk group. Interestingly, CRG-Score low risk group also showed higher T cell co-inhibition and APC co-inhibition scores (Figure 5B). The CRG-Score low risk group coexists with a state of immunosuppression and a potential immune escape mechanism.

Subsequently, we further analyzed the top 15 mutated genes between the CRG-Score risk groups (Figure 5C). TTN, OBSCN, MUC16, RYR2, CSMD3, and FBXW7 have higher mutation frequencies in the CRG-Score low risk group. At the same time, KRAS and TP53 have higher mutation frequencies in the CRG-Score high risk group. These mutations may be associated with hyperimmune infiltration (Hu and Sun, 2018; Li et al., 2020; Liu et al., 2021; Lu et al., 2021; Xu et al., 2021; Shen et al., 2022; Yang et al., 2022). These conclusions need further exploration and validation.

Due to significantly different mutation frequencies between CRG-Score risk groups, we further assessed TMB between CRG-Score risk groups. TMB was statistically different between different CRG-Score risk groups (*p* = 0.039, Figure 5D). The CRG-Score low risk group has higher TMB scores. The current literature has confirmed that TMB will bring stronger immunogenicity to tumor tissue (McGranahan et al., 2016). High TMB tumors associated with longer survival after immune checkpoint inhibitor therapy (Valero et al., 2021).

Finally, expression levels of 47 immune checkpoint-related genes were analyzed between CRG-Score risk groups (*p* < 0.05, Figure 5E). Except for TNFRSF25 and ADORA2A, the other 16 immune checkpoints were highly expressed in CRG-Score low risk group (Figure 5E). Taken together, CRC patients with low CRG scores may be better candidates for immunotherapy.

### Drug susceptibility prediction and the illustration

**FIGURE 6:**
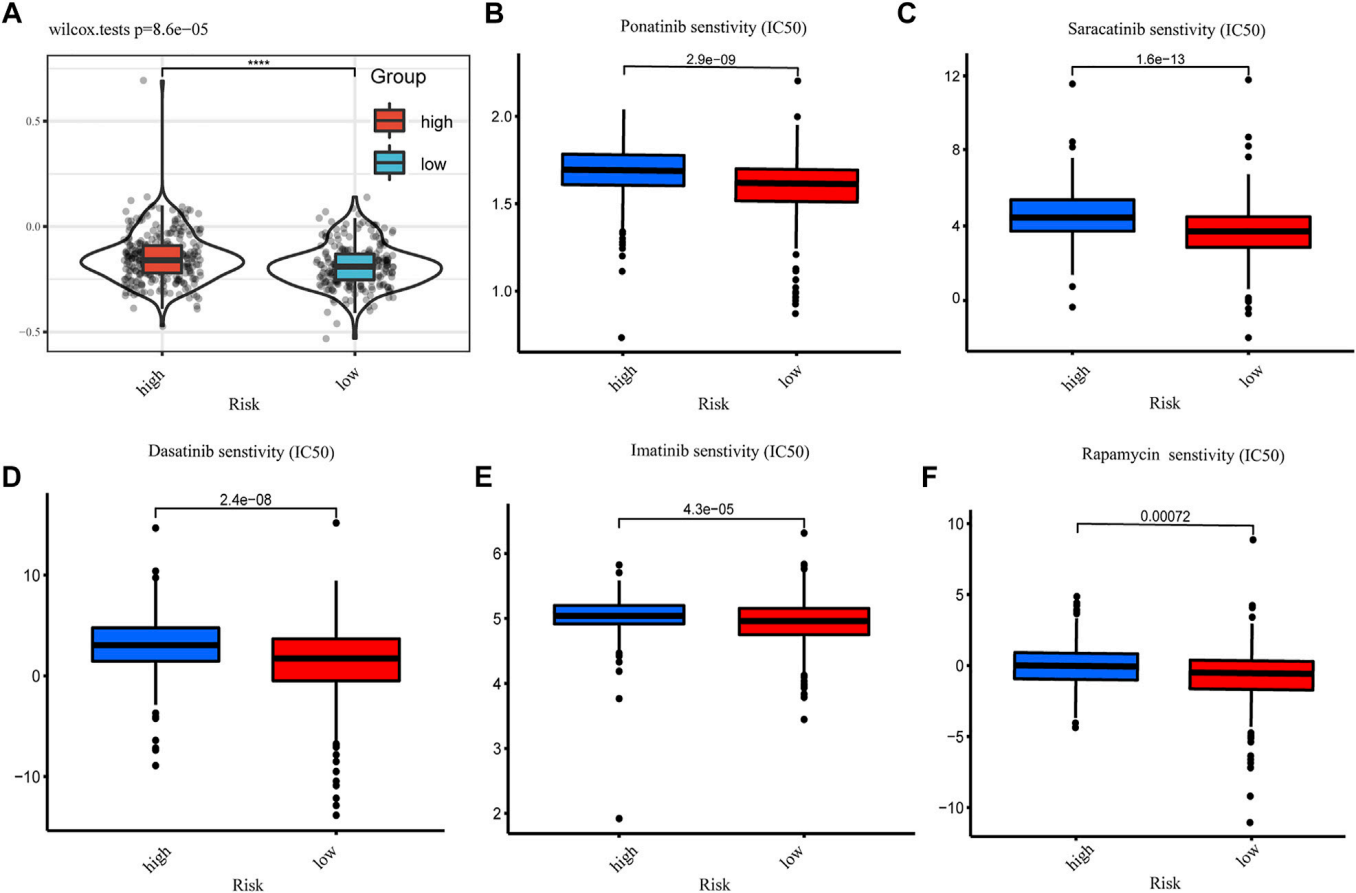
Drug susceptibility prediction. **(A)** Tumor immune dysfunction and exclusion (TIDE) scores between CRG-Score groups (*p < 0.05, **p < 0.01, ***p < 0.001). **(B)** IC50 values of Ponatinib between CRG-Score groups. **(C)** IC50 values of Saracatinib between CRG-Score groups. **(D)** IC50 values of Dasatinib between CRG-Score groups. **(E)** IC50 values of Imatinib between CRG-Score groups. **(F)** IC50 values of Rapamycin between CRG-Score groups.

Data on clinical response to immune checkpoint inhibitors in CRC are lacking. To correlate CRG-Score with guiding individual treatment practices, we used the TIDE website to predict immunotherapy efficacy between CRG-Score risk groups. The results showed that CRG-Score was positively correlated with TIDE score (*p* < 0.001, Figure 6A). CRC patients with low CRG scores has a higher TIDE score. They may be better candidates for immunotherapy. We also attempted to correlate the CRG-Score with the efficacy of common CRC chemotherapeutics and targeted drugs, looking for drugs that may be more sensitive to the CRG-Score high risk group. CRC patients with high CRG scores may be more sensitive to Ponatinib, Saracatinib, Dasatinib, Imatinib, and Rapamycin (Figures 6B–F).

**FIGURE 7:**
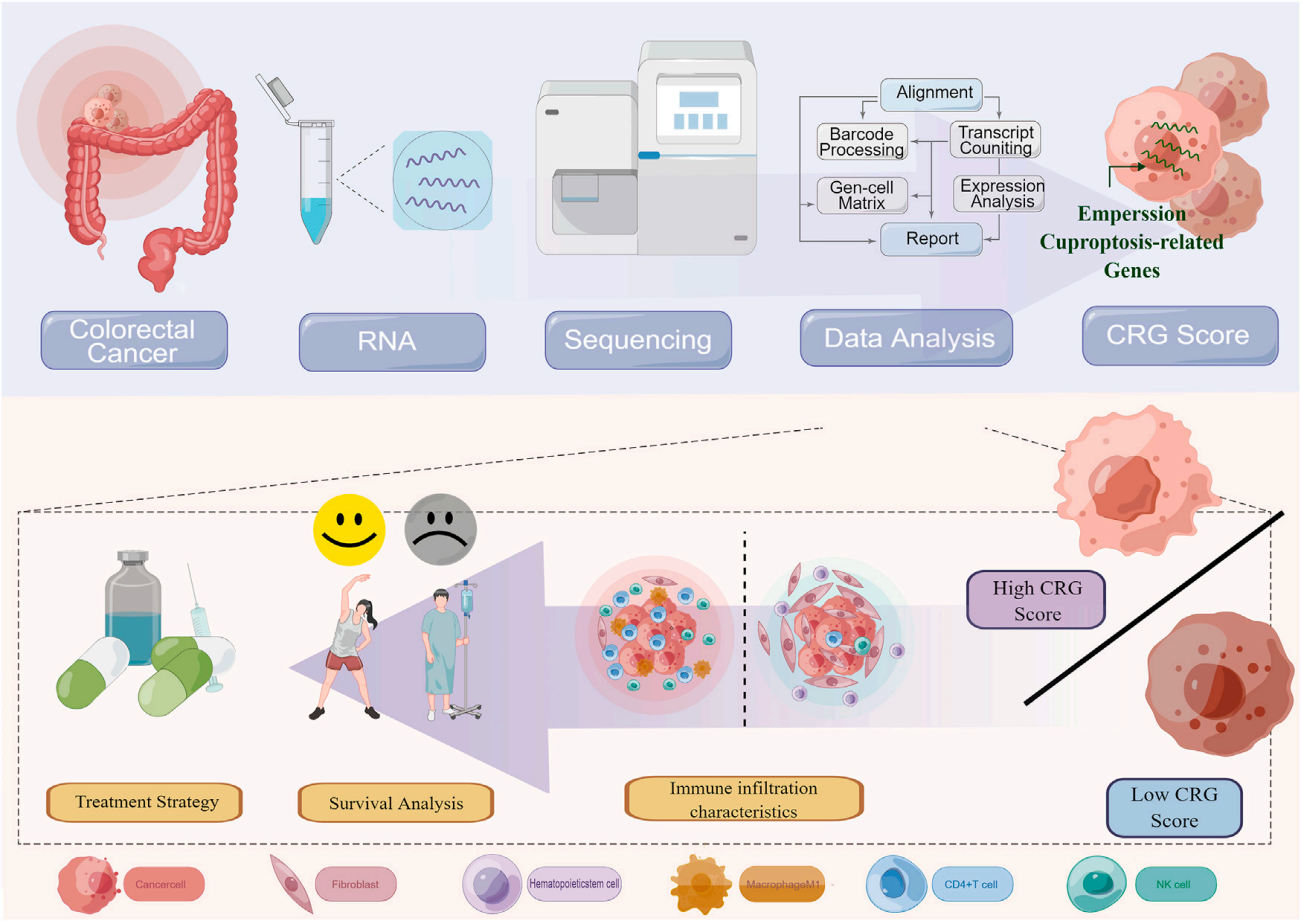
An illustration of this study.

An illustration of this study is shown in Figure 7.

## Discussion

In this study, we comprehensively assessed the landscape of 10 CRGs in CRC tissue based on TCGA. Differential expression and genetic alterations of CRGs in CRC were determined. CRG is highly correlated with initiation, progression, prognosis, and immune infiltration of CRC. We construct a risk score signature containing 3 CRGs. The nomogram integrating the CRG-Score has robust predictive performance. CRG-Score can effectively predict the immune landscape of CRC patients. Low-risk CRC patients have greater immunogenicity and higher immune checkpoint expression. Low-risk CRC patients may be better candidates for immunotherapy. At the same time, we also predicted more sensitive drugs in the high-risk CRC patients.

Evidence from a new study shows that CRG is a prognostic molecular marker for kidney cancer (Bian et al., 2022). But their effect in CRC remains unknown. To our surprise, most CRGs were differentially expressed between tumor tissues and adjacent non-tumor tissues. CRGs was strongly associated with important initiation and progression mechanisms of CRC. In univariate Cox regression analysis, 2 CRGs (CDKN2A, DLAT) were significantly associated with OS. The expression level of most CRGs strongly correlated with the level of immune cell infiltration. These results hint us that CRG may play a potential role in CRC and the possibility of using CRG to build a prognostic model.

The CRG risk score signature consists of 3 CRGs (CDKN2A, DLAT, GLS). CDKN2A can induce cell cycle arrest in G1 and G2 phases. It is closely related to a variety of tumors (Bartels et al., 2018; Adib et al., 2021; Luan et al., 2021). Dihydrolipoamide S-acetyltransferase (DLAT), a component of the pyruvate dehydrogenase (PDH) complex, catalyzes the overall conversion of pyruvate to CO2 and acetyl-CoA, thereby linking the glycolytic pathway to TCA cycle is linked. Copper binds to proteins containing fatty acylated structures in the TCA cycle can lead to aberrant oligomerization of DLAT (21). GLS is a glutaminase that converts glutamine to glutamate. Cells convert glutamine to glutamate. Glutamate is converted to alpha-ketoglutarate by glutamate dehydrogenase (GLUD) or a group of transaminases. The converted *α*-ketoglutarate enters the TCA cycle (DeBerardinis and Cheng, 2010; Michalak et al., 2015). GLS has been shown to promote tumor cell growth by modulating cell metabolism (Herranz et al., 2015; Zhang et al., 2019; Mukha et al., 2021; Tong et al., 2021).

Cuproptosis is a new cell death mechanism that relies on copper regulation. Copper binds to proteins containing fatty acylated structures in the tricarboxylic acid (TCA) cycle, resulting in abnormal aggregation of the latter and loss of iron-sulfur cluster proteins, triggering proteotoxic stress and eventual cell death (Tsvetkov et al., 2022). This may provide a new strategy for using copper toxicity to treat tumors. Based on different CRG-Score, we divided into two risk groups. It was unexpectedly found to be closely related to tumor immunity.

The CRG-Score low risk group have higher immune infiltration status and immune-related functional scores. These results suggest that it has higher immunogenicity. We further analyzed the top 15 mutated genes between the CRG-Score risk groups. TTN, OBSCN, MUC16, RYR2, CSMD3, and FBXW7 have higher mutation frequencies in the CRG-Score low risk group. At the same time, KRAS and TP53 have lower mutation frequencies in the CRG-Score low risk group. Reported so far: CRC patients with double TTN/OBSCN mutations were significantly associated with high immune infiltration and the “immune-hot” subtype (Liu et al., 2021). MUC16 mutations can enhance the infiltration of cytotoxic T lymphocytes to enhance antitumor immunity in patients with endometrial cancer (Hu and Sun, 2018). RYR2 is frequently mutated in breast cancer, and its mutations can enhance the infiltration of cytotoxic T lymphocytes, activate memory CD4^+^ T cells and M1 macrophages to enhance antitumor immune responses (Xu et al., 2021). CSMD3 mutations may promote the transformation of M0 macrophages to M2 macrophages, while leading to increased CD8^+^ T cell infiltration (Lu et al., 2021). FBXW7 mutations stimulate IFNα/β, CXCL9/10 and antigen presentation machinery by promoting EYA2 degradation, resulting in increased infiltration of cytotoxic T and NK cells (Shen et al., 2022). TP53-mutated cancers have significantly lower antitumor immune signature levels than TP53-wildtype cancers in CRC (Li et al., 2020). KRAS mutations drive immunosuppression and immunotherapy resistance in colorectal cancer through the IRF2-CXCL3-CXCR2 axis (Yang et al., 2022). The above reports suggest that these genes mutations may be associated with higher immunogenicity in the CRG-Score low risk group. However, these conclusions need further exploration and validation.

Due to significantly different mutation frequencies between CRG-Score risk groups, we further assessed TMB between CRG-Score risk groups. TMB was statistically different between different CRG-Score risk groups. TMB is currently considered to be able to predict the efficacy of immune checkpoint inhibitor drugs, and can play a predictive value as a biomarker for a variety of malignant tumors (Chan et al., 2019; Lapke et al., 2021). Malignant tumors with high TMB are usually accompanied by better immunotherapy response (Chan et al., 2019; Lapke et al., 2021). However, our results showed that the low-risk group had higher TMB scores. These suggest that our score may reflect the response to immunotherapy to a certain extent. Immune checkpoint molecules play a vital role in tumor immune escape (Schreiber et al., 2011; Noguchi et al., 2017). 16/47 immune checkpoint-related genes were differentially expressed between risk groups. We can regroup CRC patients based on CRG-Score patterns and select appropriate immune checkpoint inhibitors.

Data on clinical response to immune checkpoint inhibitors in CRC are lacking. To correlate CRG-Score with guiding individual treatment practices, we used the TIDE website to predict immunotherapy efficacy between CRG-Score risk groups. CRC patients with low CRG scores has a higher TIDE score. They may be better candidates for immunotherapy. Finally, based on IC50 values, we predicted common CRC drug sensitivities in different CRGs score groups. CRC patients with high CRG scores may be more sensitive to Ponatinib, Saracatinib, Dasatinib, Imatinib, and Rapamycin. These findings suggest that the CRG-Score has predictive value in individualizing treatment selection in CRC.

Our study has several limitations. First, our research data is based on retrospective data from public databases, lacking large-scale, prospective, real-world data for validation. Secondly, our research also lacks molecular biology support, and in-depth basic experiments are needed in the future. Finally, it should be emphasized that the low CRG scores may be more sensitive to immunotherapy in our study. However, due to the lack of cohort data on CRC immunotherapy and the strong heterogeneity among tumors, more evidence is needed to confirm our conclusions.

In conclusion, we constructed a CRG risk score signature to predict the prognosis of CRC patients. Patients with low CRG-Score lived longer. Our findings provide an immune landscape of CRC patients with different CRG-Score. The CRG-Score can be used to stratify patients and provide strategies for individual treatment.

## Data Availability

The original contributions presented in the study are included in the article/Supplementary Material, further inquiries can be directed to the corresponding authors.

## Supplementary material

SUPPLEMENTARY FIGURE S1 Further verification of the CRG risk score signature. 844 CRC data from GEO database(GSE17538,GSE29623,GSE39582) were integrated.**(A)** Heatmap of the expression of 3 CRGs between CRG-Score groups. **(B)** CRG-Score distribution between CRG-Score groups. **(C)** CRG-Score survival status between CRG-Score groups. **(D)** Survival time between CRG-Score groups. **(E)** ROC curve validated validated the prognostic performance of CRG-Score.
