# Supplementary figures and images for: Cuproptosis patterns and tumor immune infiltration characterization in colorectal cancer
Source: Front Genet. 2022 Sep 13;13:976007. doi: 10.3389/fgene.2022.976007 (PMC9513614; doi:10.3389/fgene.2022.976007)

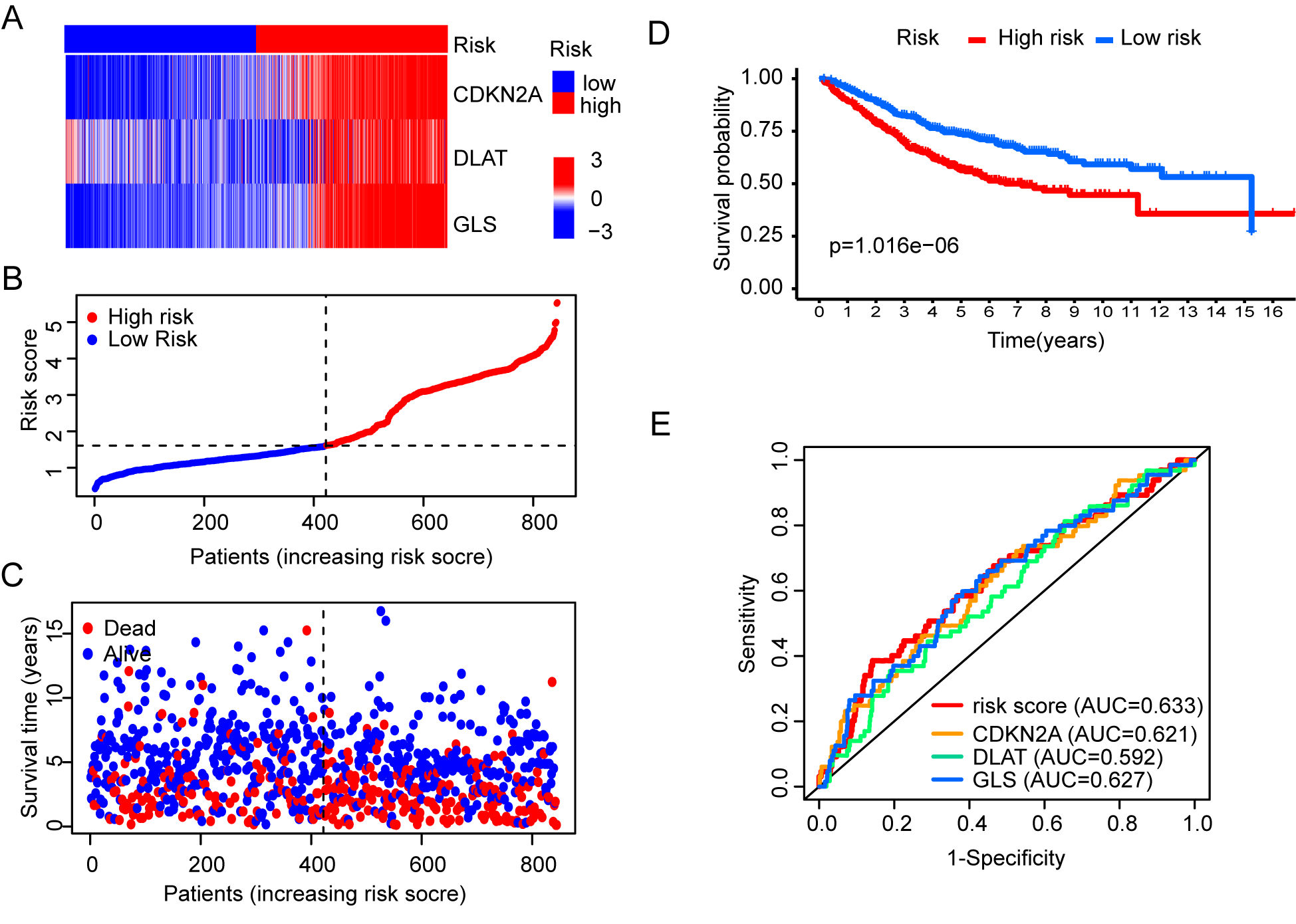

Supplement: Supplementary file 2 [file Image1.tif]
