# Supplementary material for: Cuproptosis patterns and tumor immune infiltration characterization in colorectal cancer
Source: Front Genet. 2022 Sep 13;13:976007. doi: 10.3389/fgene.2022.976007 (PMC9513614; doi:10.3389/fgene.2022.976007)
Supplement: Supplementary file 4 [file Table3.docx]

**TableS3.** Clinical information of the high CRG-Score and low CRG-Score groups.

| Covariates | High CRG-Score | Low CRG-Score | *P* value |
| --- | --- | --- | --- |
| Age |  |  | 0.2477 |
| age≤65 | 123(41.14%) | 112(46.47%) |  |
| age>65 | 176(58.86%) | 129(53.53%) |  |
| Gender |  |  | 0.5338 |
| FEMALE | 136(45.48%) | 117(48.55%) |  |
| MALE | 163(54.52%) | 124(51.45%) |  |
| Stage |  |  | 0.0389 |
| I | 45(15.05%) | 48(19.92%) |  |
| II | 106(35.45%) | 101(41.91%) |  |
| III | 91(30.43%) | 57(23.65%) |  |
| IV | 50(16.72%) | 27(11.2%) |  |
| unknown | 7(2.34%) | 8(3.32%) |  |
| T stage |  |  | 0.0318 |
| T1 | 4(1.34%) | 11(4.56%) |  |
| T2 | 48(16.05%) | 45(18.67%) |  |
| T3 | 203(67.89%) | 165(68.46%) |  |
| T4 | 43(14.38%) | 20(8.3%) |  |
| Tis | 1(0.33%) | 0(0%) |  |
| N stage |  |  | 0.0019 |
| N0 | 157(52.51%) | 160(66.39%) |  |
| N1 | 78(26.09%) | 51(21.16%) |  |
| N2 | 64(21.4%) | 29(12.03%) |  |
| unknown | 0(0%) | 1(0.41%) |  |
| M stage |  |  | 0.114 |
| M0 | 216(72.24%) | 185(76.76%) |  |
| M1 | 49(16.39%) | 27(11.2%) |  |
| unknown | 34(11.37%) | 29(12.03%) |  |
